# An *in* *vitro* agent-based modelling approach to optimization of culture medium for generating muscle cells

**DOI:** 10.1098/rsif.2023.0603

**Published:** 2024-01-17

**Authors:** David Hardman, Katharina Hennig, Edgar R. Gomes, William Roman, Miguel O. Bernabeu

**Affiliations:** ^1^ Centre for Medical Informatics, Usher Institute, The University of Edinburgh, Edinburgh EH16 4UX, UK; ^2^ Instituto de Medicina Molecular, Faculdade de Medicina, Universidade de Lisboa, Avenida Professor Egas Moniz, 1649-028 Lisboa, Portugal; ^3^ Australian Regenerative Medicine Institute, Monash University, Clayton, Australia; ^4^ The Bayes Centre, University of Edinburgh, Edinburgh EH8 9BT, UK

**Keywords:** muscle, agent-based model, tissue engineering, nuclei, computational biology

## Abstract

Methodologies for culturing muscle tissue are currently lacking in terms of quality and quantity of mature cells produced. We analyse images from *in vitro* experiments to quantify the effects of culture media composition on mouse-derived myoblast behaviour and myotube quality. Metrics of early indicators of cell quality were defined. Images of muscle cell differentiation reveal that altering culture media significantly affects quality indicators and myoblast migratory behaviours. To study the effects of early-stage cell behaviours on mature cell quality, metrics drawn from experimental images or inferred by approximate Bayesian computation (ABC) were applied as inputs to an agent-based model (ABM) of skeletal muscle cell differentiation with quality indicator metrics as outputs. Computational modelling was used to inform further *in vitro* experiments to predict the optimum media composition for culturing muscle cells. Our results suggest that myonuclei production in myotubes is inversely related to early-stage nuclei fusion index and that myonuclei density and spatial distribution are correlated with residence time of fusing myoblasts, the age at which myotube–myotube fusion ends and the repulsion force between myonuclei. Culture media with 5% serum was found to produce the optimum cell quality and to make muscle cells cultured in a neuron differentiation medium viable.

## 1. Introduction

A major challenge in the engineering of functional skeletal muscle tissue is the inability to reproduce the complex *in vivo* microenvironment of muscle tissue *in vitro* [1]. To form contractile myotubes, distinct cell sources are required. Currently, the most relevant *in vitro* myogenesis model employs primary myoblasts [2] and a tailored culture medium to successfully differentiate myoblasts into multi-nucleated myotubes.

Successful *in vitro* myogenesis is dependent upon cell culture medium composition and requires it to be fine-tuned to produce healthy and mature myotubes. Since *in vivo* myogenesis relies on several cell types, it is also important to ensure that the designed medium is compatible with these other cell types for muscle co-cultures.

Consideration of the media conditions that facilitate optimal muscle cell formation is necessary to maximize the quality, yield and reproducibility while reducing costs and experimental time. Relying on experimental trial and error to generate more sophisticated cell-based *in vitro* systems is impracticable and new strategies are necessary for culture medium optimization [3]. *In vitro* experiments are invaluable tools for exploring cell culture environments [4], but they remain expensive, time consuming and provide sparse data points for analysis. Numerical models of cell and tissue behaviours [5] present fast, low-cost methods for simulating *in vitro* experiments but require thorough calibration against experimental results to establish confidence in predictions. Given the lengthy duration of time required to produce mature myotubes *in vitro*, the ability to predict outcomes of cell experiments from early-stage indicators of cell behaviour would save experimental time and costs. Finding a single early-stage behaviour which is predictive of mature cell quality would allow direct inference of cell outcomes. Agent-based models (ABM) apply simple sets of behavioural rules to autonomous agents (such as cells or cell nuclei) and simulate their interactions with each other and their environment in order to model higher level emerging behaviours. ABM have been used to complement cell and tissue engineering as a method to gain insight into the underlying mechanisms [6], but to our knowledge have not directly been applied to the challenge of optimization of cell culture environments. Studies using ABM generally derive agent rules from the literature [7]. Here, we present a workflow for a combined *in vitro–in silico* approach to determining muscle cell quality in which experimental data are used to define metrics of muscle cell quality and of early-stage cell behaviours. Metrics of early-stage cell behaviours are applied as inputs to a nuclei-based ABM with two phases, the first modelling myoblast motion and fusion, and the second the nuclei force balance within myotube cells with metrics of cell quality indicators as an output.

We selected serum concentration as the first media variable since primary myoblast fusion into multi-nucleated myotubes occurs upon serum reduction [8] but the cellular effects of varying serum at these low concentrations remain ill-defined. We chose the proportion of a neuronal differentiation media as our second component. Co-culturing muscle cells with neurons increases muscle cell maturation through neuronal-derived factors [9] and provides the possibility to study neuromuscular junction (NMJ) formation, and so it is of interest to study the extent to which high-quality myotube production is achievable in media tailored to neuron differentiation.

Healthy muscle cells are long multi-nucleated cells with evenly distributed nuclei throughout the periphery of the fibre. Since uneven or centralized distribution of nuclei is often linked to a disease state or improper maturation [10,11], nuclear position and distribution is a good marker of muscle cell maturity and health while the proportion of myoblasts fusing to become myonuclei informs us of the efficiency of cell differentiation. We therefore chose nuclei positions of both unfused myoblasts and fused myotubes (myonuclei) as the agents in our ABM. Nuclei are also suited to the role of agent here as their motion can be tracked over time from images, enabling quantification of their behaviour. This enables us to calibrate a model linking nuclear behaviour with cell health.

Key metrics of cell behaviours are measured from *in vitro* imaging and, for behaviours which we were unable to measure directly, inferred using an approximate Bayesian computation sequential Monte Carlo (ABC–SMC) method. A sensitivity analysis is performed to determine the effects of both measured and inferred cell behaviours on cell quality indicators. Early-stage behaviour metrics found to correlate with cell quality indicators are fitted to functions of differentiation media composition from which we can predict media conditions favourable for cell growth. These functions are used to inform the media composition of a further *in vitro* experiment in order to more accurately map the optimum conditions for culturing muscle cells.

## 2. Results

### 2.1. Changes in differentiation medium composition affect both early behaviour and quality indicators

Generating bottom-up cell-based systems relies on the timely development of cells. Cellular behaviours early in the culture will therefore govern the culture’s quality at later stages. As such, we used live and fixed imaging to identify a list of potential indicators divided into early-stage behaviours and quality indicators (table 1). To be of use in a predictive model, indicators must show significant variation in relation to one or more of the cell culture variables being optimized.

**Table 1.** List of metrics

indicator	metric
early-stage behaviours	myoblast speed (*S*_*mb*_)
	myoblast variation in angular velocity (*ω*_*mb*_)
	myoblast rate of proliferation (*P*)
	myoblast angular velocity
	myoblast direction of motion
	myoblast persistence of motion
	myoblast velocity
cell quality indicators	difference in myonuclei density (days 0–5) (*dM*)
	myonuclei coefficient of variation in spatial distribution (*D*_var_)
	mean distance between myonuclei (*D*)
	myotube width
	proportion myotubes with actin striations

**Figure 1.**
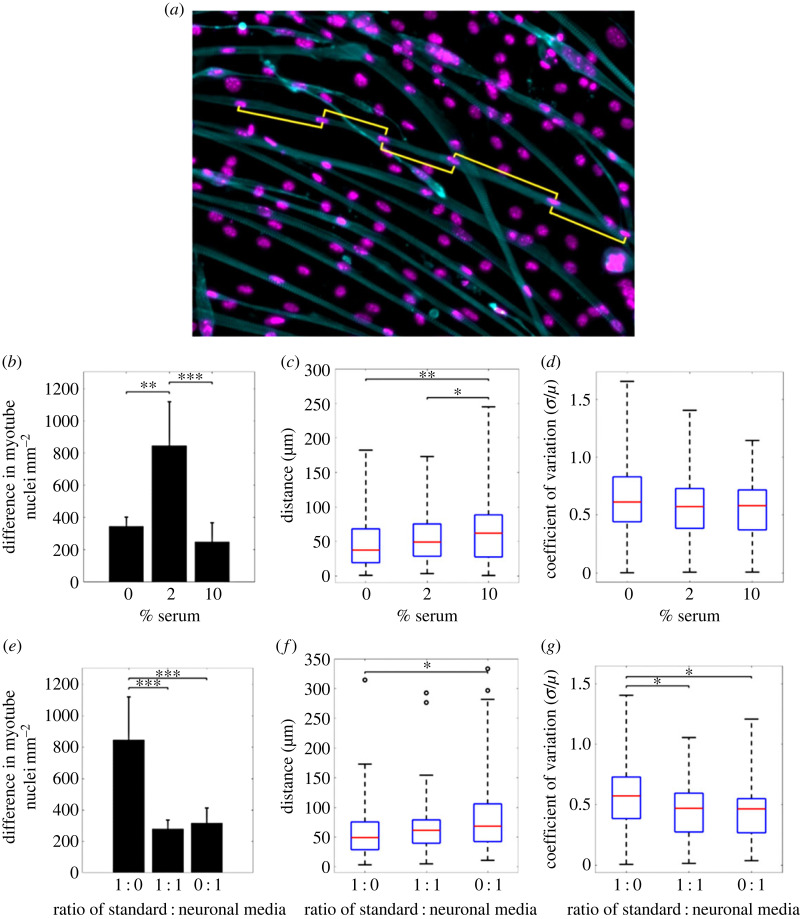
Myotube quality indicators at day 5 of differentiation. (*a*) A fixed image stained for cell nuclei (magenta) and actin cytoskeleton (cyan), yellow lines indicate measured distances between myonuclei. Plots (*b*-*d*) show differences in quality indicators with varying serum concentrations. (*b*) Difference in myonuclei $\mathrm{per}\;{\mathrm{mm}}^{2}$ between days 0 and 5 of differentiation, (*c*) mean distance between myonuclei and (*d*) uniformity of distance between myonuclei expressed as ‘coefficient of variation in spatial distribution’. Plots (*e*–*g*) show differences in quality indicators in varying ratios of neuronal medium. (*e*) Difference in myonuclei $\mathrm{per}\;{\mathrm{mm}}^{2}$, (*f*) mean distance between myonuclei and (*g*) coefficient of variation. In (*b*) and (*e*), values are reported as mean ± s.d.

Potential indicators of mature cell quality were extracted from fixed, stained images (figure 1*a*).

Increasing the serum concentration (figure 1*b*) from 0% to 2% led to a significant (464 more myonuclei mm^−2^) increase in the difference in density of myonuclei *dM* for cells cultured in muscle cell differentiation media. A further increase to 10% serum concentration resulted in a reduction in *dM* (559 fewer myonuclei mm^−2^) to a level slightly lower than with no serum. Increasing the concentration of serum resulted in a gradual increase in the mean distance between myonuclei (*D*) (figure 1*c*) but, there was no significant change in coefficient of variation in myonuclei distribution (*D*_var_) (figure 1*d*).

A significant drop in *dM* (567 less myonuclei mm^−2^) was observed between experiments cultured in muscle cell differentiation media and in muscle and neuronal cell differentiation media mixed in a 1 : 1 ratio (figure 1*e*). *dM* remained at a similarly low level in experiments with 100% neuronal medium, demonstrating the existence of an optimal base medium for myotube formation. Increasing the proportion of neuronal medium led to an increase in *D* (figure 1*f*). Experiments with 100% neuronal medium and a 1 : 1 mixture of muscle and neuronal cell differentiation media exhibited a lower *D*_var_ , and therefore more uniform distribution, than muscle cell differentiation medium alone (figure 1*g*).

Mean thickness of myotube cells (electronic supplementary material, figure S1) and proportion of sampled myotubes with full or partial actin striations (electronic supplementary material, figure S2) were also studied as potential quality indicators, but did not display significant differences between trials with different media compositions and so were discounted as appropriate quality indicators for our workflow.

**Figure 2.**
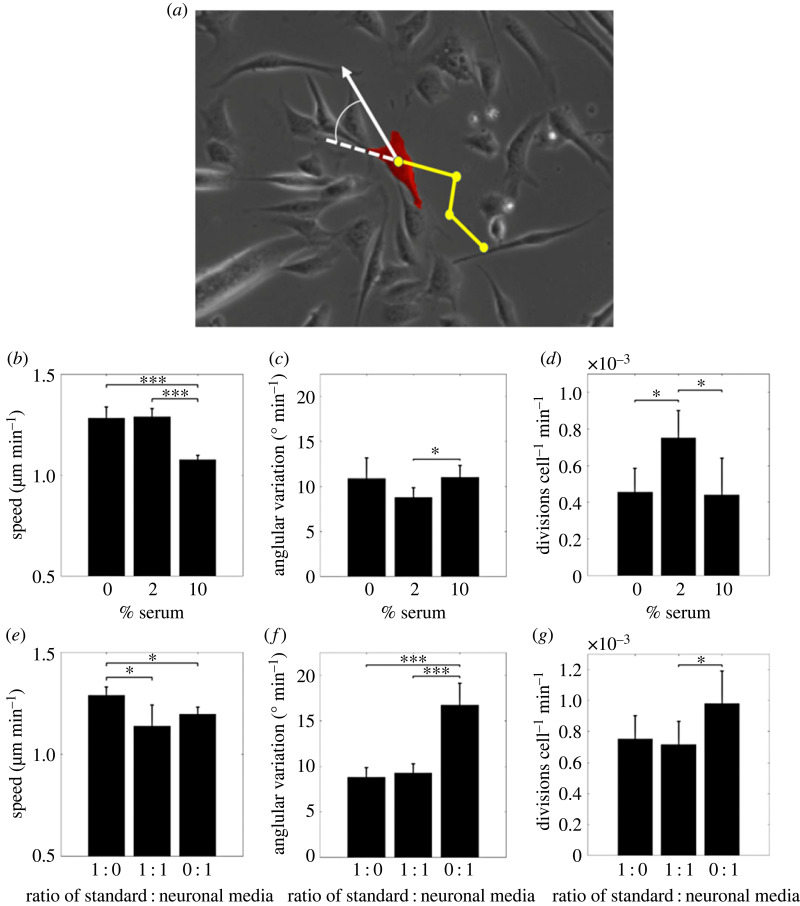
Metrics of myoblast behaviour from live images of cells. (*a*) Illustration of tracking a single myoblast (red) in a brightfield image. Yellow dots show position in previous frames. Plots (*b*–*d*) show differences in early-stage behaviour metrics with varying serum concentrations. Myoblast (*b*) speed, (*c*) variation in angular velocity and (*d*) rate of proliferation. Plots (*e*–*g*) show differences in behaviour metrics in varying ratios of neuronal medium and 2% serum concentration. Myoblast (*e*) speed, (*f*) variation in angular velocity and (*g*) rate of proliferation. In (*b*–*f*) values are reported as mean ± s.d.

To create metrics of early-stage cell behaviours, myoblast cells were segmented and tracked from live images taken during days 0–1 after the application of differentiation media (figure 2*a*). Significant and nonlinear differences in metrics of myoblast speed of motion *S*_*mb*_, myoblast variation in angular velocity (*ω*_*mb*_) and myoblast proliferation rate (*P*) were observed.

For serum concentrations between 0 and 2%, myoblast cells moved with similar *S*_*mb*_ (figure 2*b*). Increasing serum concentration from 2 to 10% resulted in a significant reduction in *S*_*mb*_. There was also evidence of a modest dip in *ω*_*mb*_ as serum concentration is increased from 0 to 10% (figure 2*c*). *P* increased significantly at a serum concentration from 2% (figure 2*d*).

Concerning distinct muscle-neuron media mixtures, we observed a decrease in *S*_*mb*_ when muscle cell differentiation medium was replaced with neuronal differentiation medium or mixed to 1 : 1 ratios (figure 2*e*). However, *ω*_*mb*_ (figure 2*f*) and *P* (figure 2*g*) were only altered when muscle cell differentiation medium was fully replaced with neuronal differentiation medium.

Mean direction and persistence of myoblast motion were also extracted from tracking cells in live images. On average, cells in all images analysed displayed no global directional preference and so mean direction was discounted as an early-stage behavioural indicator.

### 2.2. A calibrated agent-based model predicts indicators of cell quality from *in vitro* early-stage cell behaviours measurements

While our results show that both early behaviour and quality indicators vary with differentiation media composition, we found no direct relationship between any single early behaviour and the quality of mature myotubes. To study whether mature cell quality can be determined from the interaction between early-stage behaviours, we designed an ABM of myoblast fusion and myonuclei migration to simulate the complex interactions between early-stage behaviours and produce metrics of quality indicators as an output. The ABM is described in detail in the electronic supplementary material, Methods.

Calibration of the residence time mechanism and force-balance acting on fused nuclei requires the input of further metrics of cell behaviours which were not directly measurable from our imaging data (see table 4 in Methods). The use of approximate Bayesian computation (ABC) allows us to infer distributions of these metrics by strategically running ABM simulations and comparing their outputs with measured quality indicator metrics. Two extra *in vitro* trials were conducted to provide conditions with further combinations of serum and neuronal differentiation medium. One trial with 5% serum and muscle and neuronal differentiation medium mixed in a ratio of 1 : 4 and the other with 10% serum and exclusively neuronal differentiation medium.

**Figure 3.**
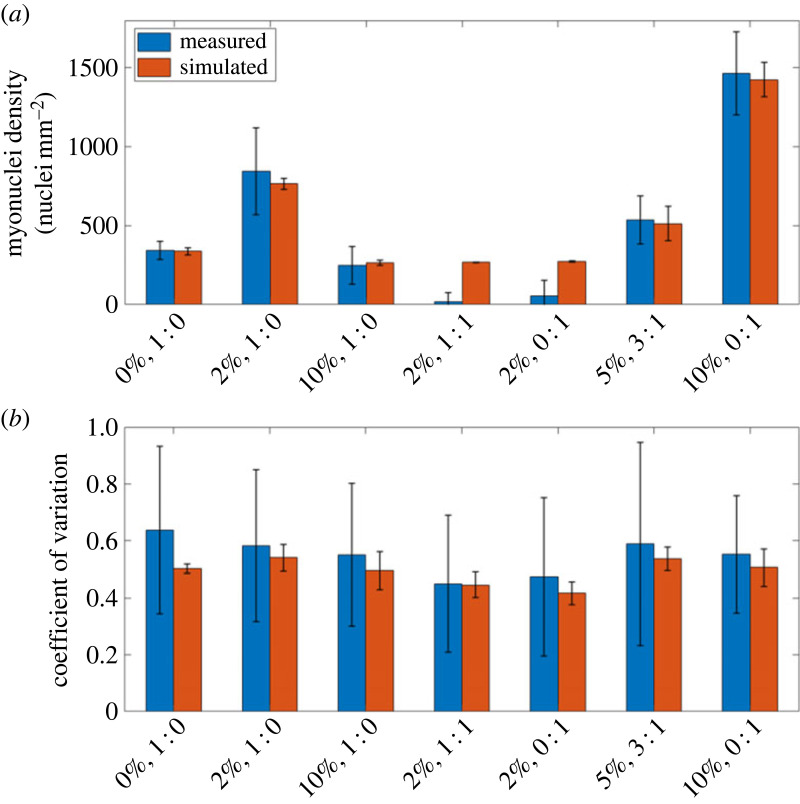
Comparison of measured and simulated quality indicators at day 5 of differentiation. Myonuclei quality metrics measured from imaging data from *in vitro* trials against outputs from the calibrated agent-based model for varying serum concentrations and ratios of muscle to neuronal medium. (*a*) Myonuclei density. (*b*) Coefficient of variation in spatial distribution, a metric of nuclei spatial uniformity. Values are reported as mean ± s.d.

ABM simulations reproduced mean myonuclei densities to within 1 s.d. of those observed experimentally for most conditions (figure 3*a*). The exceptions of 2% serum with 1 : 1 muscle to neuronal cell differentiation media and 2% serum with 100% neuronal cell differentiation medium, which exhibit day 5 myonuclei densities significantly smaller than the initial number of myoblasts. The ABM assumes that myoblasts do not die during the experimental timeframe and all have the potential to fuse throughout the differentiation stage and so the total number of nuclei at day 0 is the minimum number of myonuclei obtainable from simulation.

To assess whether the ABM simulations successfully reproduce changes in quality indicators over time, the mean myonuclei densities in an *in vitro* 2% serum and no neuronal medium trial were measured on each day of differentiation and compared with simulations. Simulated myonuclei density (electronic supplementary material, figure S3) was found to be within 1 s.d. with measured for each day.

Median values of *D*_var_ were reproduced by the ABM to within 1 s.d. for all trials (figure 3*b* and electronic supplementary material, table S1). The ABM results showed a smaller *D*_var_ than measurements from imaging data (figure 3*b* and electronic supplementary material, table S1). ABM simulations also reproduced measurements of *D* from imaging data to within 1 s.d. (electronic supplementary material, table S1).

These results imply that the *in vitro*/ABM workflow outlined here is sufficiently calibrated to reproduce average values of quality indicators in trials in which fusion occurs.

### 2.3. Cell behaviour parameters inferred via approximate Bayesian computation are strongly related to cell quality indicators

**Figure 4.**
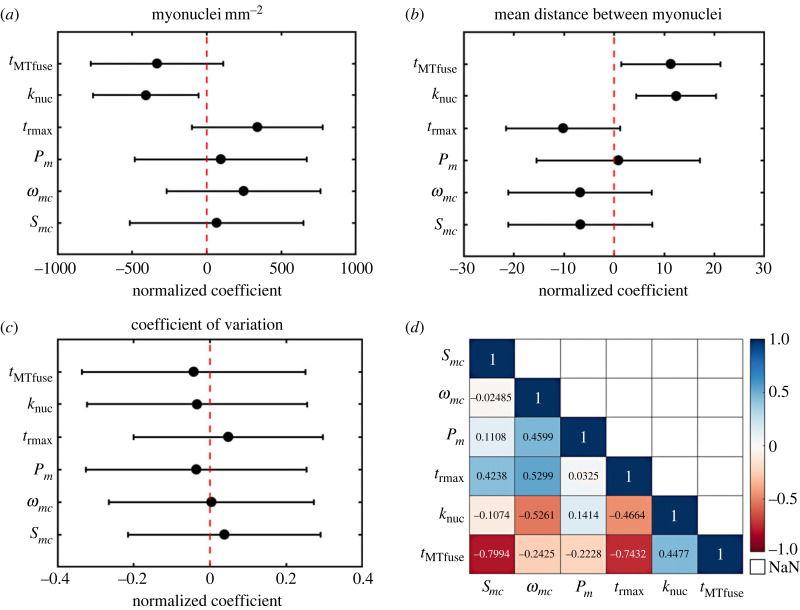
Linear regression analysis of measured and inferred cell behaviour parameters against cell outcomes from *in vitro* experiments. Normalized coefficients of cell behaviour parameters with 95% confidence intervals against (*a*) myonuclei mm^−2^, (*b*) mean distance between myonuclei in myotubes and (*c*) coefficient of variation of myonuclei spatial distribution, a measure of spatial uniformity.

A set of linear regression analyses of measured and inferred cell behaviour metrics against cell quality indicators from *in vitro* experiments was performed to determine the relative effect size of each metric. The nuclei repulsion force constant (*k*_nuc_) was estimated to have a significant effect (*t* = −2.97, *p* < 0.05) on difference in myonuclei mm^−^^2^ (figure 4*a*), with smaller *k*_nuc_ relating to higher densities of myonuclei. Myotube–myotube fusion time threshold (*t*_MTfuse_) and the residence time threshold (*t*_rmax_) were estimated to have some, though not significant (*t* = 1.93, *p* = 0.11 and *t* = 1.98, *p* = 0.10, respectively), effect on *dM* while *S*_*mb*_ and *P* were estimated to have the least effect (*t* = 0.29, *p* = 0.78 and *t* = 0.42, *p* = 0.69, respectively).

*k*_nuc_ and *t*_MTfuse_ were positively associated with (*t* = 3.11, *p* = 0.01 and *t* = 2.93, *p* = 0.03, respectively) *D* (figure 4*b*). *t*_rmax_ was weakly negatively related to (*t* = −2.30, *p* = 0.07) *D*, while *ω*_*mc*_ and *S*_*mb*_ have a limited negative relation (*t* = −1.22, *p* = 0.28 and *t* = −1.21, *p* = 0.28) and *P*_*m*_ was estimated to have the least effect (*t* = 0.13, *p* = 0.90). Owing to the large variability in *D*_var_ , all the cell behaviour metrics were estimated to have limited to no effect (figure 4*c*). The linear regression analysis shows that all of the cell behaviours which were estimated to be significantly associated with cell outcomes and quality indicators are inferred rather than measured parameters. A comparison of correlation between cell behaviour parameters (figure 4*d*) shows a significant (*p* = 0.03) negative correlation between *S*_*mb*_ and *t*_MTfuse_. There is also some, non-significant (*p* = 0.06), negative correlation between *t*_rmax_ and *t*_MTfuse_.

### 2.4. Fitting functions of inferred cell behaviours from discrete *in vitro* experimental data informs media composition of future experiments

**Figure 5.**
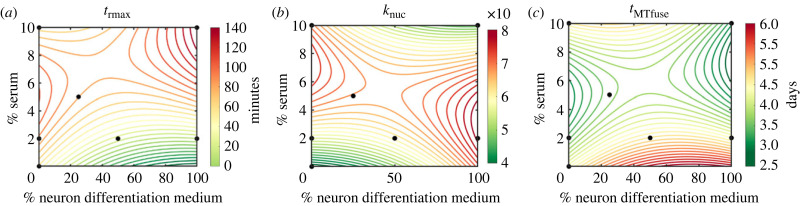
Fitted surface functions for inferred cell behaviours from *in vitro* imaging data. Second-order fit of behavioural parameters. (*a*) Residence time threshold, (*b*) nuclei repulsion force coefficient and (*c*) maximum age at which myotubes can fuse. Black dots denote media compositions of *in vitro* trials.

Having determined the significant effects of early-stage cell behaviours inferred via the ABC–SMC method on cell quality indicators, we describe how these key behaviours change with respect to the composition of the differentiation media in order to suggest optimal media compositions for future *in vitro* experiments. The effects of media composition on cell behaviours are nonlinear and so a second-degree polynomial surface model was applied to fit metrics of cell behaviours from each of the previous *in vitro* trials (figure 5).

Increasing serum concentration was shown to correspond with an increase in *t*_rmax_ (figure 5*a*) suggesting that, at higher serum concentrations, myoblasts require a longer time in contact with myotubes before fusion can occur. *k*_nuc_ (figure 5*b*) showed a general increase with increased serum concentrations, with a slight decline at high concentrations of serum (greater than approx. 7%). For media with lower serum concentrations (less than approx. 4%), *k*_nuc_ increases as the concentration of neuronal differentiation medium is increased. *t*_MTfuse_ was projected to be between 3 and 4.5 days of cell differentiation for most media conditions (figure 5*c*). This agrees with observations of actin striations, an indicator of cell maturity, occurring in cells after day 3. *t*_MTfuse_ was shown to be earlier in cells cultured in an increased proportion of neuronal differentiation medium and continue for longer in cells cultured in higher serum concentrations. Since simulations were ended at day 5, cells in a medium with no neuronal differentiation medium and 10% (or greater) serum concentration may still allow fusion at day 5 and beyond. The surface plots indicate a local maxima for *t*_rmax_ and a local minima for *t*_MTfuse_ at a media composition of 5% serum and 0% N2B27 differentiation media and so this composition was chosen for a further exploratory *in vitro* trial (*k*_nuc_ is also predicted to be relatively high at this composition, though the nearest local maxima is at 7% serum).

### 2.5. Serum concentrations of 5% predicted to be optimal in media with low neuron differentiation media concentrations

**Figure 6.**
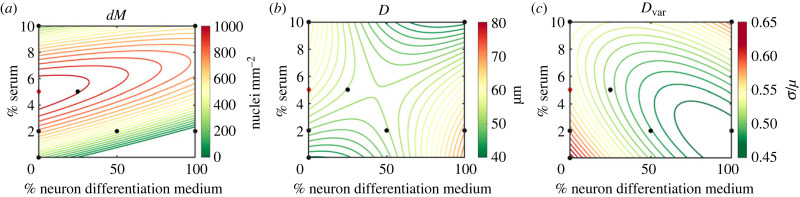
Fitted surface functions for cell quality indicator metrics *in vitro* imaging data. Second-order fit of metrics of (*a*) myonuclei mm^−^^2^, (*b*) mean distance between myonuclei in myotubes and (*c*) coefficient of variation of myonuclei spatial distribution. Black dots denote media compositions of *in vitro* trials. Red diamond denotes extra *in vitro* trial informed from analysis of inferred cell behaviours.

Fitting second-order surface functions to quality indicator metrics from *in vitro* trials, including the additional trial at 5% serum and no N2B27 medium as suggested by analysis of inferred cell behaviours, allows visualization of the effects of varying media composition and the prediction of an optimal media composition for culturing primary muscle cells (figure 6). Increasing the concentration of serum is predicted to increase the *dM*, indicating increased fusion (figure 6). Above 5–6% serum, *dM* then begins to diminish. Increasing the concentration of neuron differentiation medium was shown to inhibit fusion (*dM* tends to zero), especially in media with low serum concentrations. In media with no serum, no fusion is predicted above an N2B27 concentration of 24% though the addition of serum concentrations over 2% fusion is predicted to be initiated even in media with 100% neuron differentiation media. An increase in serum concentration is predicted to result in a gradual increase in *D* when N2B27 differentiation media concentrations are below 50% (figure 6). This trend is inverted in N2B27 concentrations above 50%, with the largest *D* predicted to be in media with no serum and 100% N2B27 differentiation media. These findings fit the trends observed in *k*_nuc_ (figure 6) and so *D* in media with low serum and high N2B27 concentrations will be influenced by both larger *k*_nuc_ and little to no increase in *dM* due to lack of fusion. In media with less than 50% neuron differentiation media, increasing serum concentrations from 0 to 5% is predicted to decrease *D*_var_ in myotubes indicating increased uniformity in distribution. At high concentrations of N2B27 this was reversed, and higher serum concentrations predict less uniformly distributed myonuclei.

Our results indicate that a high concentration of neuron differentiation medium has a detrimental effect on muscle cell fusion, though this can be reversed through the addition of higher concentrations of serum to the differentiation media. For media in 100% muscle differentiation media, we predict the optimum serum concentration to be 5%. This aligns with the maximum predicted *dM*, and lower concentrations result in less uniformly distributed nuclei, while higher concentrations have no significant effect on uniformity of distribution. This optimum media composition fits with predictions made from the inferred early-stage behaviours *t*_rmax_ and *t*_MTfuse_.

### 2.6. Difference in total myonuclei production is inversely related to early-stage nuclei fusion index

**Figure 7.**
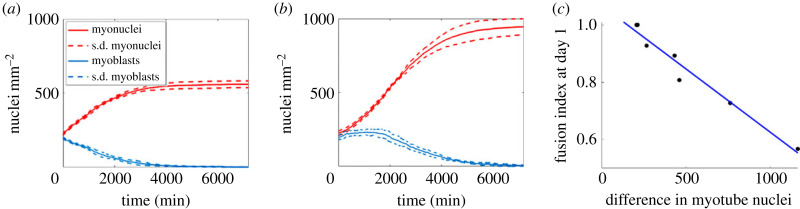
Relation between increase in myonuclei density and fusion index. Examples of agent-based model (ABM) generated changes in myonuclei (red) and myoblast (blue) nuclei density over time for conditions (*a*) 0% serum in neuronal medium and (*b*) 2% serum in neuronal medium (dashed lines represent standard deviation over five runs). (*c*) ABM generated differences in number of myonuclei between days 0 and 5 against fusion index (*R*^2^ = 0.96).

One advantage of ABM methods compared with using regression models alone is the insight they give into the possible mechanisms which affect the outcomes of a model. From ABM simulations throughout the parameter space, it was noted that conditions generating small (300–400 nuclei mm^−^^2^) total increases in *dM* (figure 7*a*) exhibit a negative exponential curve in the increase in myonuclei density over time and a steep initial decline in myoblast numbers, while larger total increases in myonuclei (figure 7*b*) are associated with a sigmoidal increase in myonuclei over time with nuclei initially increasing or remaining stable before decreasing. To account for these distinctive trends, we first compared the rate of myonuclei production between days 0 and 1 with day 5 difference in myonuclei for all media-type experiments but found no significant correlation.

Further analysis indicates that these differences in total number of myonuclei (and therefore the number of fusion events) relate to the ratio of myonuclei to total nuclei, described by the nuclei fusion index [12], during the initial stages of differentiation. There is a strong negative linear relation (*R*^2^ = 0.96) between *dM* and the nuclei fusion index at day 1 (figure 7*c*).

### 2.7. Computational modelling for all conditions can be completed in less than half the time required for a single *in vitro* trial

To evaluate the time saved using our workflow compared with non-computational methods of optimization, we break down the typical time taken to perform each step of the workflow. These timings are summarized in table 2. Computational modelling in the workflow is designed for parallel computing and so the time taken to complete tasks will vary significantly depending upon the computational resources available. We assume that the *in vitro* trials required for alternative optimization methods are at least as extensive as those proposed in our workflow. See Methods for further details.

**Table 2.** Time taken to complete key workflow tasks.

task	breakdown of timings	total time	in parallel/additional to *in vitro* trials
acquisition of live images	2 h/video for seven trials.	14 h	in parallel
analysis of live images	5 min/video for six positions and 30 min processing data for seven trials	7 h	in parallel
cell division count	10 min/video for six positions in seven trials	7 h	in parallel
ABM–ABC computation of inferred behaviours	20 min/simulation for up to 300 simulations run in parallel on 16 cores for seven trials	43.75 h	additional
ABM parameter sweep.	20 min/simulation for 121 simulations run in parallel on 16 cores	2.5 h	additional
total time	—	74.3 h	—
total extra time	—	46.3 h	—

A parameter sweep of 121 discrete media compositions was achieved in under 3 h using a 16-core processor to run simulations in parallel. In total, the live image acquisition and analysis and computational effort required to implement this workflow was less than 3.5 days, of which up to 28 h was run in parallel with *in vitro* cell maturation. For comparison, a single *in vitro* trial requires 9 days to reach day 5 of myotube differentiation (4 days in growth medium and 5 days in differentiation medium).

## 3. Discussion

Optimization of cell culturing protocols in tissue engineering is essential for maintaining the quantity and functionality of mature cells as well as ensuring experimental reproducibility. Applying quantitative mathematical and computational modelling approaches alongside *in vitro* experiments increases efficiency and reduces costs when designing cell culturing procedures, aiding the transition from bench to bedside for regenerative medicine [13].

We developed novel metrics for indicating the quality and quantity of mature muscle cells cultured from murine myoblasts and measured metrics of migratory and proliferative myoblast behaviour. We found that varying the concentrations of media components produces significant changes in indicators of mature muscle cell quality and key early-stage cell behaviours. We created and calibrated an agent-based model (ABM) to estimate indicators of cell quality using metrics of early-stage behaviour as inputs. Using outputs of the ABM and *in vitro* measurements, we inferred further early-stage metrics of cell fusion and nuclei movement within myotubes. These inferred metrics were determined to have significant effects on indicators of muscle cell quality and were applied to define the media composition of a further *in vitro* trial in order to predict optimum muscle cell growth. Serum is added to media to stabilize conditions for cell proliferation, though differentiation in some muscle cell lines has been shown to be triggered by the deprivation of serum. Serum represents an unknown in terms of composition, which may alter motor neuron differentiation efficiency and trigger loss of stemness. Our analysis indicates that, while muscle cell differentiation is viable in media without serum, applying moderate levels of serum boosts the amount of fusion as well as increasing both the spread and the uniformity of myonuclei. Previous work by Lawson & Purslow [8] shows C2C12 cells differentiated best in low serum media, and other studies [14] show using media with low serum is advantageous in preparation for co-culturing muscle cells with iPS-derived motor neurons. Our work indicates that the relationship between serum concentration and cell fusion is nonlinear. The addition of moderate concentrations of serum (up to around 5%) has significant benefits to cell growth without impacting cell quality, with these benefits diminishing at higher concentrations. Co-culturing muscle cells with neurons has been shown to increase muscle cell maturation [9] and the ability to culture muscle and neuronal cells in parallel would be a step towards recreating an *in vivo* environment for muscle cells. To achieve this, a culture medium which effectively sustains and differentiates both muscle and neuronal cells is required. Our results show that increasing the concentration of a neuron differentiation medium inhibits fusion of myoblasts, though this effect could be partially reversed through the addition of higher serum concentrations. Though cell fusion was decreased with higher concentrations of neuron differentiation medium, there was no significant decrease in cell quality. Uniform distribution of myonuclei is known to be associated with cells in healthy muscle tissue [15], with aggregation of nuclei linked to muscular disfunction [10,16,17]. In this study, the mean distance between myonuclei and coefficient of variation of myonuclei were applied as metrics of the uniformity of myonuclei distribution within a cell. The difference in myonuclei density per mm^2^ over the first 5 days of differentiation was used as a second indicator of cell outcomes. Trials showing greater increases in myonuclei density at the end of the differentiation phase exhibited a lower fusion index during the early stages of differentiation. This appears counterintuitive, but since our simulations indicate that myoblast availability is a limiting factor in cell fusion rate, we hypothesize that media compositions with a lower fusion index prioritize myoblast proliferation over fusion during the early stages of differentiation. The supply of myoblasts will therefore not be exhausted as quickly as more fusion-efficient regimes, leading to a greater total amount of fusion. The negative relation between fusion index and total amount of fusion has wider implications for the reliance of fusion index alone as a marker of experimental success. As fusion index measures the ratio of myonuclei to mononucleated myoblasts at a given time, it conflates the rate of cell–cell fusion with the proliferation rate of myoblasts. While fusion index is simple to calculate and gives an intuition of initial experimental efficiency, it is important to consider that a high fusion index may be due to a high fusion rate of neighbouring cells or a low rate of background cell division, or some more complex balance between the two. Since availability of myoblasts appears to be a limiting factor in the total amount of fusion, and thus the final volume of cells, it may seem intuitive to culture cells to confluence during the growth phase so there is greater availability. Previous studies [18], however, show that seeding myoblasts at a high density induces quiescence in cells and suppresses differentiation and so would not provide an effective strategy. Committed myoblasts need to switch from a proliferation state (growth phase) to a fusion state (differentiation phase). In *in vitro* culture, this switch can be triggered by lowering media serum content, which causes myoblasts to initiate cell cycle arrest and differentiate into myotubes, but this behaviour may be strongly altered if medium composition and serum concentration are not optimal.

Our numerical modelling assumes that metrics of cell motion do not change over time. It may be expected that cell migration speed and angular velocity change as myotube density increases with time, though we found no significant changes in metrics of behaviour (results not shown) throughout the first day of differentiation. Temporal changes over a longer timescale were not studied but assumed to have a minor impact on model outputs due to the short timespan (1–3 days) in which the bulk of fusion occurs. Our model also assumes that all myoblasts may differentiate into myotubes. This may not always be the case, for instance C2C12 cells which are deficient in the production of the proteins Myf-5 and Myo-D have been shown to fail to form myotubes in culture [19]. The low myonuclei densities observed here for high levels of neuron differentiation media indicate that not all myoblasts are viable, or that significant numbers of myoblasts die before they can fuse. We hypothesize that the assumption of universal myoblast differentiation or the lack of a cell death mechanism (or both) within the ABM lead to an over-prediction of myotube formation by the ABM in experiments in which myonuclei densities are significantly smaller than the initial number of myoblasts. Nuclei stainings which differentiate between myoblasts and myonuclei would allow a clearer observation of myoblast fates and allow for the inclusion of model parameters to determine myoblast viability of differentiation and death. Additional early markers of cell quality include myotube width and the presence of actin striations in myotubes [20]. We found no significant differences in width and a high proportion of actin striations present in all trials and so did not include them in our investigation, though they may be relevant for optimizing other cell culture protocols or cell lines. Current practices in cell culturing tend to apply a trial-and-error approach to experimental design, though there is a call to move to more systematic methods of optimization [5]. Our workflow of informing *in vitro* experiments by quantifying images and analysing *in silico* experiments can be extended as an iterative process to further optimize cell culture conditions. It also provides an insight into the mechanisms underlying the changes in cell quality which is absent in methods relying purely on regression analysis. The workflow described here is designed to be applicable to other cell types, providing they exhibit early behaviours and quality indicators which can be quantified and that mature cell quality is not correlated with a single behavioural indicator. In this study, we optimize just two cell culture conditions, but once behavioural and quality indicators have been acquired, adding further variables for optimization is trivial. The number of trials required for optimization using a design of experiment (*DoE*) method increase exponentially with increasing numbers of factors observed and so the application of a workflow for reducing the number of *in vitro* trials will become increasingly more cost-effective. Factors for optimization include further media compositions, materials for substrate structure and topological constraints such as patternings for aligning cells. The design and calibration of the ABM from scratch is the most time consuming element of our workflow and requires specialist skills which may not be immediately available to labs. We have designed the ABM to be both user-friendly and customizable for general application, using open-source software packages where possible. NetLogo software for ABM design provides a user-friendly graphical user interface (GUI). To obtain inferred cell behaviours, NetLogo is linked to PyABC via the PyNetLogo extension, example code available on Github, (https://github.com/dhardma2/MyoChip). PyABC features provision for extensive parallel computing for this task and so a calculation of the time required for calibration will depend upon the number processors available. By switching off the cell fusion mechanism, our ABM can be applied as a generic model of motile cell behaviour which could be used as a base for building similar predictive models of further cell types. While the fusion dynamics of the ABM could have potential in studying the formation of further types of syncytia, a more obvious application is the optimization of further skeletal muscle cell lines including human-induced pluripotent stem cells and the study of muscular degenerative diseases.

In summary, we identified new quality indicators for muscle cells, which can be implemented in future statistical approaches to optimization. We showed that changes in the composition of muscle cell differentiation media affect both early-stage cell migratory behaviours, cell fusion index and cell quality. We designed an ABM to study the effects of measured and inferred early-stage cell behaviours on cell quality. Our results indicate that moderate concentrations of serum can boost both the quantity and quality of muscle cells and can prevent the inhibitory effect of neuron differentiation medium on myoblast fusion. Extending our workflow to an iterative set of *in vitro–in silico* experiments here would allow optimization of a wide range of further muscle cell culturing parameters and muscle progenitor cell lines, including human iPS, providing an efficient and cost-effective method for improving and quantifying tissue engineering procedures.

## 4. Methods

### 4.1. *In vitro* primary myotube culture

*In vitro* myotubes were cultured from primary mice myoblasts as described previously [21]. Shortly, hind limb muscles were isolated from 5- to 7-day-old mice pups, minced and digested for 1.5 h at 37°C using 0.5 mg ml^−1^ collagenase (Sigma) and 3.5 mg ml^−1^ dispase (Roche) in phosphate-buffered saline (PBS). Subsequently, filtered cell suspension was plated in IMDM (Invitrogen) for 4 h in the incubator (37°C, 5% CO_2_). To purify cells from fibroblasts and other contaminating cell types, only non-adherent myoblasts were collected and centrifuged. Cells were resuspended in growth medium (IMDM+20% FBS +1% ChickEmbryoExtract+1% penicillin/streptomycin) and plated onto 1 : 100 matrigel (RD) coated ibidi dishes. After 4 days, medium was changed to trigger differentiation. Depending on the experimental condition, standard muscle differentiation medium (IMDM+1% penicillin/streptomycin) and neuronal medium (N2B27 medium: 50% DMEM-F12, 50% Neurobasal+1XN2+1XB27+50 μM β-Mercaptoethanol + 1% penicillin/streptomycin) were mixed 1 : 0, 1 : 1 or 0 : 1, containing 0, 2, 5 or 10% horse serum. After 1 day of differentiation, a thick layer of 1 : 1 matrigel was added on top of forming myotubes and 100 ng ml^−1^ agrin was added to the culture medium. Cells were cultured up to 7 days at $37^{\circ }C/5\%\;{\mathrm{CO}}_{2}$.

The Rodent Facility of the Instituto de Medicina Molecular João Lobo Antunes maintains high standards of animal welfare and promotes a responsible use of animals, hence supporting state-of-the-art animal-based research. The Rodent Facility has a license of establishment for breeding and animal use, issued by the Portuguese competent authority—Direcção-Geral de Alimentação e Veterinária (DGAV)—since 2013. This facility complies with Portuguese law Decreto-Lei 113/2013, transposed from the European Directive 2010/63/EU, and follows the European Commission recommendations (2007/526/EC) on housing and care of animals and the FELASA (Federation of European Laboratory Animal Science Associations) guidelines concerning laboratory animal welfare. (Ethics approval number AEC 2014 03 EG).

### 4.2. Live imaging

Live imaging was performed using an inverted microscope (Zeiss Cell Observer SD or Zeiss Cell observer) in widefield mode, using a 20× phase air objective (Plan-Apochromat Ph2 NA 0.80 or EC Plan-Neofluar Ph 2 NA 0.50, respectively). Cells were imaged 2 h after adding the respective differentiation medium; 3 × 3 tiles with 10% overlap with 2 × 2 binning were taken every 5 min for a minimum of 12 h. Images were stitched using Zen Blue software and stacked into 30 min videos for further quantitative analysis.

### 4.3. Immunostaining and static imaging

Cells were washed once with PBS and fixed in 4% PFA for 10 min at room temperature. Cells were permeabilized (PBS + 0.5% Triton) for 5 min and blocked in blocking buffer (10% in goat serum in PBS + 5% BSA) for 1 hour at room temperature. Primary antibodies (1 : 200; Anti-*α*-Actinin (Sarcomeric) mouse monoclonal antibody, Sigma Aldrich and A7732) were diluted in blocking buffer containing 0.1% saponine and cells were incubated at 4°C over night. Dishes were washed twice for 5 min in PBS under agitation. Secondary antibodies (1 : 400; Goat anti-Mouse IgG Alexa Fluor 555, Thermo Fisher Scientific, A21424; Goat anti-Rabbit IgG Alexa Fluor 647, Thermo Fisher Scientific, A21245) in blocking buffer were incubated in the presence of DAPI (100 μg ml^−1^) for 1 h. Dishes were washed twice, 200 μl Fluoromount-G (Invitrogen) was added on top of cells. Samples were stored at 4°C. Dishes were imaged with an inverted fluorescent microscope (Zeiss Cell observer) using a 40× phase air objective (EC Plan-NeoFluar NA 0.75). *z*-stacks of 1 μm were taken as 3 × 3 tiles with 10% overlap and 1 × 1 binning. Subsequently, images were stitched in Zen Blue.

### 4.4. Agent-based model

NetLogo software [22]. NetLogo. (http://ccl.northwestern.edu/netlogo/. Center for Connected Learning and Computer-Based Modeling, Northwestern University. Evanston, IL.) was used to create a nuclei-centred agent-based model (ABM). Code is available on Github (https://github.com/dhardma2/MyoChip). A pseudocode description outlining the rules of the ABM is illustrated in electronic supplementary material, figure S5. Terms and abbreviations used are shown in table 3.

**Table 3.** List of terms and abbreviations.

name	description	symbol
agent-based model	computational model simulating actions and interactions of autonomous agents. Here, agents are cell nuclei	ABM
approximate Bayesian computation–sequential Monte Carlo method	method for estimating posterior distributions of model parameters without a likelihood function	ABC–SMC
myoblast	single nuclei muscle precursor cell	
myotube	multi-nucleated cell produced from fused myoblasts	
myonuclei	nuclei of a myotube cell	
quality indicators	measurable early indicators of eventual muscle cell quality and quantity. Here, we use coefficient of variance of myonuclei distance and change in myonuclei density	*Q*
early-stage behaviours	metrics of myoblast behaviour which vary significantly with culture media composition, applied as inputs to the ABM. Here, we use myoblast speed, angular velocity and proliferation rate	*B*
media function	general term for function determining quality indicators for a given media parameter	*M*(*x*)
proportion of neuronal differentiation medium	the fraction of differentiation medium comprising neuron specific N2B27 medium	*α*
serum concentration	percentage of serum by volume in cell culture media	*β*
myonuclei distance	mean distance between myonuclei in given sample cell	*D*
myonuclei coefficient of variation in spatial distribution	quality indicator of uniformity in myonuclei distribution ($D_{\sigma }/\bar{D}$)	*D*_var_
difference in myonuclei density	quality indicator of cell fusion events, measured as difference in myonuclei mm^−^^2^ between days 0 and 5 of differentiation	*dM*
myoblast speed	mean product of distance travelled by a myoblast cell between frames and frame rate (μm min^−1^)	*S*_*mb*_
myoblast variation in angular velocity	standard deviation of angular deviation of myoblast motion between frames multiplied by frame rate (degrees min^−1^)	*ω*_*mb*_
myoblast rate of proliferation	cell divisions per cell min^−1^	*P*
area of influence	area surrounding nuclei in which the residence time counter is activated if another nuclei is detected	AoI
residence time threshold	the maximum time that two nuclei may spend within each other’s AoI before fusion is initiated	*t*_rmax_
combined residence time parameter	AoI/*t*_rmax_	*γ*
myotube–myotube fusion time threshold	the maximum age that a fused nuclei can initiate fusion with another myonuclei	*t*_MTfuse_
coefficient of nuclei lateral force	coefficient governing the distance-dependent lateral force enacting on myonuclei	*k*_lat_
coefficient of nuclei repulsion force	coefficient of myonuclei repulsion force, simulating the actin-driven lengthwise repulsion of nuclei	*k*_nuc_

**Figure 8.**
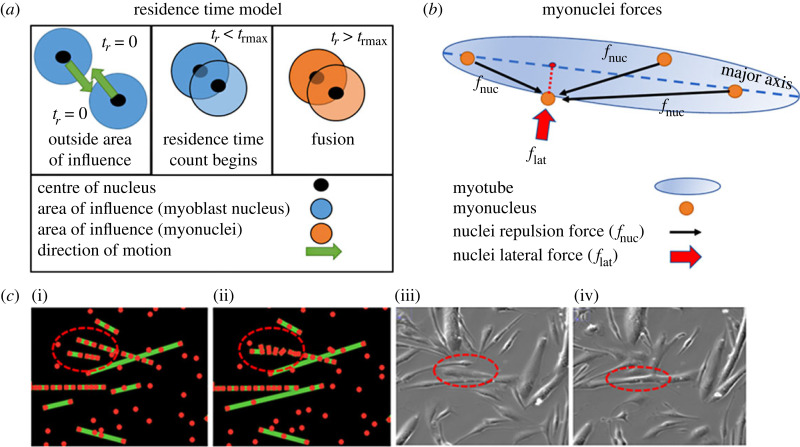
Overview of agent-based model. (*a*) Residence time model of myoblast–myotube fusion. *t*_rmax_ is threshold residence time. (*b*) Representation of forces acting on a single nucleus. A lateral force (*f*_lat_) moves the nucleus towards the major axis of nuclei in a myotube in a direction normal to the major axis. Total nuclei repulsion force is the sum of the individual repulsion forces (*f*_nuc_) acting on the nucleus. (*c*) Myotube–myotube fusion in agent-based model at (i) 0 min and (ii) 15 min (red dots represent nuclei and green lines represent myotubes) and in live images at (iii) 0 min and (iv) 15 min.

The ABM is seeded with initial densities of myoblast nuclei and myonuclei. Random sampling from normal distributions of measured and inferred myoblast behaviour metrics is used to apply the required inputs to model myoblast motion. Myoblast–myotube and myotube–myotube fusion is modelled via a residence time model. Once fused, a force balance is applied to myonuclei to simulate lateral and longitudinal motion within the cell. The ABM provides metrics of the densities of myoblast nuclei and myonuclei as well as distances between nuclei within discrete myotubes. Figure 8 presents an overview of the ABM methodology. A detailed description of the initialization, modelling of myoblast motion, cell fusion, myonuclei motion and outputs of the ABM is found in the electronic supplementary material, Methods.

### 4.5. Model workflow

**Figure 9.**
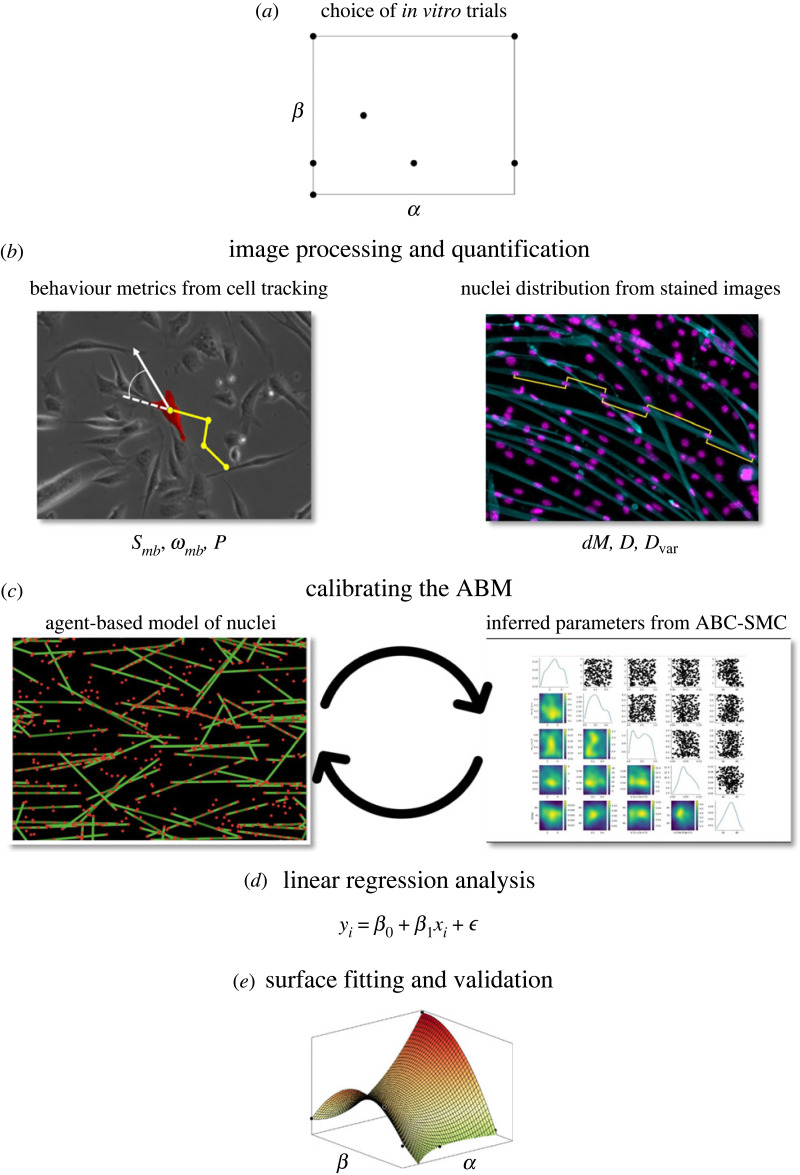
Overview of *in* *vitro*–*in* *silico* workflow. (*a*) Conduct initial *in vitro* trials with discrete concentrations of serum and neuronal differentiation medium. (*b*) Obtain measured distributions of *S*_*mb*_, *ω*_*mb*_ and *P* from tracking cells in live images. Measure *dM*, *D* and *D*_var_ = *σ*/*D* from fixed, stained images. (*c*) Representative images of ABM (left-hand side, red dots represent nuclei and green lines represent myotubes) and visualization of posterior distributions from pyABC (right-hand side). Parameters of inferred cell behaviours derived using an ABC–SMC method. Multiple ABM runs, informed by measured myoblast behaviours, are compared with measured cell quality indicators (*Q*). (*d*) Apply linear regression analysis to estimate the effect size of measured and inferred early-stage behavioural metrics (*B*) on *Q*. (*e*) Fit second-order surfaces to values of *B* found to be predictive of *Q* to determine concentrations of serum and neuronal differentiation medium likely to produce high *Q* then conduct further *in vitro* trial(s) with suggested media compositions.

An overview of the workflow in creating a predictive model of cell quality is presented in figure 9 and a detailed description of each process is give in the electronic supplementary material, Methods.

*In vitro* experiments with different concentrations of media variables provide data with which to initialize and calibrate the ABM (figure 9*a*). Live images of myoblasts are analysed to provide metrics of early-stage cell behaviours (*B*) and fixed, stained images of myoblasts and myotubes provide cell quality indicators (*Q*) (figure 9*b*). Metrics of *B* and indicators of *Q* which vary with changes in cell culture variables are retained. If no direct relationship between any single metric *B* and indicator *Q* can be found then an ABM is employed to model the interactions between behaviours and ascertain the effect size of each metric of *B* on indicators *Q*.

Nuclei positions are the agents manipulated in the model. The main mechanisms of the ABM, chosen for their simplicity, are a residence-time cell fusion model and a force-balance model acting upon fused nuclei.

Certain parameters required to drive these mechanisms could not be measured and so, where identified, are inferred using an ABC–SMC method [23]. Observed metrics of *B* (table 4) from *in vitro* trials were applied as inputs to the ABM and multiple simulations with different values of the parameters to be inferred were run (figure 9*c*). The values of indicators *Q* produced as outputs of the ABM are compared with measured values from experimental images using a distance function based upon differences in number of fusion events, distance between myonuclei and uniformity of myonuclei distribution (see electronic supplementary material, Methods for details). ABC–SMC chooses samples of parameter values from a prior distribution and selects the samples in which the distance function output is less than a given threshold (ϵ) to build a distribution of likely values of each parameter. The size of ϵ is then systematically decreased. Probability distributions that converge towards a specific value as ϵ decreases indicate parameters which are sensitive to the indicators *Q* and qualify for further investigation. The likelihood-free methodology of ABC–SMC allows parameters to be inferred with uniformly distributed priors. The initial range of uniform prior distributions used for each inferred parameter are as follows: *t*_rmax_ from 1 to 100, *t*_MTfuse_ from 0.5 to 4.5 days, *k*_lat_ and *k*_nuc_ from 0 to 1. Further details of the ABC–SMC are described in the electronic supplementary material, Methods and summary statistics and minimum values of ϵ are found in electronic supplementary material, table S2.

**Table 4.** ABM input parameters.

model parameter	observed or inferred from data
myoblast count at *t*_0_	observed
myotube proportion at *t*_0_	observed
myoblast speed of motion (*S*_*mb*_)	observed
myoblast angular velocity (*ω*_*mb*_)	observed
myoblast rate of proliferation (*P*)	observed
nuclei area of influence/residence time threshold (*γ* = AoI/*t*_rmax_)	inferred
coefficient of lateral force (*k*_lat_)	inferred
coefficient of nuclei repulsion force (*k*_nuc_)	inferred
myotube–myotube fusion time threshold (*t*_MTfuse_)	inferred

Once the values and distributions of measured and inferred cell behaviour parameters *B* have been obtained, we apply linear regression analysis to assess the effect size of each metric *B* on indicators *Q* (figure 9*d*). If a metric of *B* is estimated to have a significant effect on one or more indicators of *Q* then the values of the metric, taken from *in vitro* trials, are fitted to a surface with axis comprising values of media composition variables (figure 9*e*). A second-degree polynomial surface model was applied as suggested for efficient response surface methodology approximation by Box & Wilson [24]. Surface plots estimate the cell media compositions which will result in local maxima and minima of each metric of *B*, which in turn relate to increases or decreases in *Q*. Taken together the plots can be used to predict media compositions which will provide the optimum values of indicators of *Q*. Once one or more predicted optimal media compositions are obtained, further *in vitro* trials can be conducted which can either be assumed to be an optimum value or used to further update the surface fitting models in an iterative fashion.

### 4.6. Evaluation of workflow timescale

Analysis of static, stained images took 2 h for each trial (5 min to download, 5 min to pre-process images and 5 min to analyse per unique position for at least six positions with 30 min to collate information). Since analysis of static images is required for a typical *in vitro* analysis, these timings have not been included here as an additional task for our proposed workflow.

Live images for each trial were acquired over a 2 h window. Automated analysis of myoblast motion behaviour for each trial took 1 h (5 min of computation per unique position for at least six positions and 30 min to collate information). Manual counting of myoblast proliferation also took 1 h (10 min per position). Live image acquisition and analysis can be run concurrently with *in vitro* trials.

A typical ABM run without the GUI takes between 10 and 20 min to simulate 5 days of cell differentiation and maturation. The ABC–SMC method requires up to 300 ABM trials to provide distributions of inferred cell behaviours. PyABC features provision for extensive parallel computing for this task and so any calculation of time required will depend upon the number of processors available. We ran pyABC on a 16-core processor, taking a maximum of 6 h 15 min to complete per trial (100 h of processing time in total). Gaining inferred behaviour distributions for the seven trials used here can therefore be achieved within a 2-day period.

Once calibrated, the ABM can then be used to predict cell quality and quantity for discrete media compositions (10–20 min per simulation) or sweep through the entire parameter space of media compositions.

### 4.7. Statistical analysis

The Wilcoxon signed-rank test was applied to assess mean distance between myonuclei, and two-sample *t*-tests were applied for all other datasets unless otherwise stated. Statistical analysis and linear regression analysis was conducted in Matlab.

## Data Availability

Imaging data used in this study is available and can be accessed from the datashare repository: https://datashare.ed.ac.uk/handle/10283/4404 [25]. Metrics extracted from image data are provided either in the main text or in the electronic supplementary material [26]. The ImageJ macros for pre-processing images, Matlab code for image analysis, the Netlogo code for the ABM and Python code for linking the ABM with pyABC are available from the Zenodo repository: https://zenodo.org/records/10300311 [27].
